# A New Remedy for Toothache

**Published:** 1848-10

**Authors:** James Beatson

**Affiliations:** Airdrie


*A New Remedy for Toothache. The Principle Agent being Chloroform.*—Mr. James Beatson, of Airdrie says,—"Gum copal, when dissolved in chloroform, forms an excellent compound for stuffing the holes of decayed teeth. I have used it frequently within the last two weeks, and the benefit which my patients have derived from it has been truly astonishing. The application is simple and easy. I clean out the hole, and moisten a little cotton with the solution; I introduce this into the decayed part, and in every instance the relief has been almost instantaneous. The chloroform removes the pain and the gum copal resists the action of the saliva, and, as the application is so agreeable, those who may labor under this dreadful malady would do well to make a trial of it."—*Ibid.*

